# Limited accuracy of conduction band effective mass equations for semiconductor quantum dots

**DOI:** 10.1038/s41598-018-21043-3

**Published:** 2018-02-13

**Authors:** Adam Mielnik-Pyszczorski, Krzysztof Gawarecki, Paweł Machnikowski

**Affiliations:** Department of Theoretical Physics, Faculty of Fundamental Problems of Technology, Wrocław University of Science and Technology, 50-370 Wrocław, Poland

## Abstract

Effective mass equations are the simplest models of carrier states in a semiconductor structures that reduce the complexity of a solid-state system to Schrödinger- or Pauli-like equations resempling those well known from quantum mechanics textbooks. Here we present a systematic derivation of a conduction-band effective mass equation for a self-assembled semiconductor quantum dot in a magnetic field from the 8-band ***k*** · ***p*** theory. The derivation allows us to classify various forms of the effective mass equations in terms of a hierarchy of approximations. We assess the accuracy of the approximations in calculating selected spectral and spin-related characteristics. We indicate the importance of preserving the off-diagonal terms of the valence band Hamiltonian and argue that an effective mass theory cannot reach satisfactory accuracy without self-consistently including non-parabolicity corrections and renormalization of ***k*** · ***p*** parameters. Quantitative comparison with the 8-band ***k*** · ***p*** results supports the phenomenological Roth-Lax-Zwerdling formula for the *g*-factor in a nanostructure.

## Introduction

The ***k*** · ***p*** method is a well-established approach to calculating the electronic and magnetic properties of bulk semiconductors^1^. It has also been applied to semiconductor nanostructures within the envelope function approximation^2,3^, in which a carrier state is described as a superposition of contributions from different bands, with local amplitudes smoothly varying in space and referred to as the *envelope wave functions*. Its accuracy is in many cases sufficient in many applications throughout mesoscopic physics, even when subtle details of the spectrum are considered^4–6^. At the same time, it offers much lower computiational costs, better scalability to larger systems and much higher transparency than the more exact atomistic approaches^7–9^. The applicability of the ***k*** · ***p*** method for modulated systems (nanostructures) has been confirmed by providing a rigorous derivation from the full Schrödinger equation^10,11^. Recent developments bring the ***k*** · ***p*** methods down to the atomistic level^12^. Currently, the 8-band ***k*** · ***p*** model is a widely tested and generally trusted standard for calculating carrier states (including magnetic effects) in mesoscopic structures, in particular in strained, self-assembled systems^13–16^.

In many cases, when the focus is on the electron states, a single-band description is desired, which would offer further reduction of computational costs, make it possible to approximate the problem by a simple, analytically solvable model, and provide still more straight-forward interpretation referring to the well-known properties of the usual Schrödinger or Pauli equations. Such models, known as *effective mass* equations, have been used for decades to describe smoothly varying perturbations of semiconductor systems, like shallow impurities^17^ and later to semiconductor heterostructures^18,19^. In the case of quantum dots (QDs), an example of a simple approach based on the effective mass approximation is the “particle in a box model”^20^ that can further be approximated by the celebrated (and analytically solvable) Fock-Darwin model^21,22^, which once offered general understanding of QD properties^23^ and is still widely used, at least as the first approximation to many problems.

In a periodic system, the reduction of the multi-band ***k*** · ***p*** model to an effective mass theory is achieved via a quasi-degenerate perturbation theory that has its roots in quantum chemistry and is referred to as Löwdin partitioning^24^. By means of this procedure, one gets for the conduction band (cb) in the lowest (second) order a Schrödinger- (or Pauli-) like equation for the envelope function with a constant effective mass and Landé factor expressed via well-known formulas in terms of the band-edge energies. Two problems are encountered in an attempt to heuristically generalize such an equation to a modulated system (a nanostructure): First, it is generally accepted that (in analogy to the multi-band Hamiltonian), the components of the wave vector ***k*** should be replaced by derivatives (momentum operators in the coordinate representation). However, at the same time, the band edges and other parameters become position-dependent via their dependence on local composition and strain. This leads to a Schrödinger-like equation with a position-dependent effective mass and Landé factor and the ordering between the momenta and these two parameters starts to play a role. This ordering is arbitrary if the effective mass model is introduced as a heuristic extension of the bulk formulas. Arguments based on solvable models of abrupt interfaces^25,26^ lead to the conclusion that the only physically correct ordering in a non-homogenous medium is the most common “*k*(1/m*)*k*”. This conclusion has been formally verified by rigorous derivation from the exact multi-band envelope function equations^2,10^.

In this work we would like to address a more practical question of the achievable quantitative accuracy of the cb effective mass theories for quantum dot (QD) systems. Thus, the goal of this paper is to rigorously derive a family of effective mass Hamiltonians for a cb electron in a strained, inhomogeneous system by applying a systematic series of approximations to the Löwdin partitioning of the 8-band ***k*** · ***p*** Hamiltonian in the envelope function approximation and to validate their predictions against the results of the 8-band model with respect to the energy spectrum and Landé factors. By *effective mass Hamiltonians* we understand cb Hamiltonians for a carrier in an external magnetic field, obtained by a unitary transformation (partitioning) that eliminates the coupling between the conduction and valence bands of the original Hamiltonian, that contain quadratic (in momentum ***k***) kinetic energy terms and may contain cubic spin-orbit couplings. We will show that reproducing the results from the 8-band theory by an effective mass equation is possible with limited precision and the form of the equation required to achieve the best available accuracy is much more complicated than the usual Schrödinger- or Pauli-like equation for the envelope function. On the other hand, the Landé *g* factor of the nanostructure can be reasonably estimated by the simple semi-phenomenological Roth-Lax-Zwerdling formula.

## The starting point: 8-band ***k*** · ***p*** model

The 8-band ***k*** · ***p*** Hamiltonian in the envelope function approximation is defined in the block notation as^3^

1 $$H=(\begin{array}{lll}{H}_{{\rm{6c6c}}} & {H}_{{\rm{6c8v}}} & {H}_{{\rm{6c7v}}}\\ {H}_{{\rm{8v6c}}} & {H}_{{\rm{8v8v}}} & {H}_{{\rm{8v7v}}}\\ {H}_{{\rm{7v6c}}} & {H}_{{\rm{7v8v}}} & {H}_{{\rm{7v7v}}}\end{array}),$$

where the blocks refer in the standard way to the cb (6c), the *j* = 3/2 valence band (vb, 8v) and the *j* = 1/2 (spin-orbit split-off) vb (7v) and are explicitly given by^3,14^

2a $${H}_{6{\rm{c}}6{\rm{c}}}={E}_{{\rm{c}}}+{V}_{{\rm{p}}}+{a}_{{\rm{c}}}\,{\rm{T}}{\rm{r}}\eta +\frac{{\hslash }^{2}}{2{m}_{0}}\,({k}_{x}{A{\rm{^{\prime} }}}_{c}{k}_{x}+\frac{i}{2}{k}_{[x}g{\rm{^{\prime} }}{k}_{y]}{\sigma }_{z}+{\rm{c}}.{\rm{p}}.)$$

2b $$\begin{array}{ccc}{H}_{8{\rm{v}}8{\rm{v}}} & = & {E}_{{\rm{v}}}-\frac{{\hslash }^{2}}{2{m}_{0}}\{{k}_{x}{\gamma {\rm{^{\prime} }}}_{1}{k}_{x}-2\,({J}_{x}^{2}-\frac{1}{3}{J}^{2})\,{k}_{x}{\gamma {\rm{^{\prime} }}}_{2}{k}_{x}\\  &  & -\{{J}_{x},{J}_{y}\}k{}_{\{x}{\gamma {\rm{^{\prime} }}}_{3}{k}_{y\}}+{\rm{c}}.{\rm{p}}.\}\\  &  & +\frac{1}{2\sqrt{3}}[\{{J}_{x},{J}_{y}^{2}-{J}_{z}^{2}\}\,\{{C}_{k},{k}_{x}\}+{\rm{c}}.{\rm{p}}.]\\  &  & +{a}_{{\rm{v}}}\,{\rm{T}}{\rm{r}}\eta -{b}_{{\rm{v}}}\,[({J}_{x}^{2}-\frac{1}{3}{J}^{2})\,{\eta }_{xx}+{\rm{c}}.{\rm{p}}.]\\  &  & -\frac{{d}_{{\rm{v}}}}{\sqrt{3}}\,[\{{J}_{x},{J}_{y}\}{\eta }_{xy}+{\rm{c}}.{\rm{p}}.]\\  &  & -i\frac{{\hslash }^{2}}{{m}_{0}}\,[k{}_{[x}\kappa {\rm{^{\prime} }}{k}_{y]}{J}_{z}+{k}_{[x}q{k}_{y]}{J}_{z}^{3}+{\rm{c}}.{\rm{p}}.],\end{array}$$

2c $$\begin{array}{ccc}{H}_{7{\rm{v}}7{\rm{v}}} & = & {E}_{{\rm{v}}}+{V}_{{\rm{p}}}-{{\rm{\Delta }}}_{0}-\frac{{\hslash }^{2}}{2{m}_{0}}({k}_{x}{{\rm{^{\prime} }}\gamma }_{1}{k}_{x}+{\rm{c}}.{\rm{p}}.)+{a}_{{\rm{v}}}\,{\rm{T}}{\rm{r}}\eta \\  &  & -i\frac{{\hslash }^{2}}{{m}_{0}}[k{}_{[x}\kappa {\rm{^{\prime} }}{k}_{y]}{\sigma }_{z}+{\rm{c}}.{\rm{p}}.]-({\mu }_{B}{B}_{z}{\sigma }_{z}+{\rm{c}}.{\rm{p}}.),\end{array}$$

2d $$\begin{array}{ccc}{H}_{6{\rm{c}}8{\rm{v}}} & = & \sqrt{3}{\boldsymbol{T}}\cdot \mathop{{\boldsymbol{k}}}\limits^{ \sim }P+i\frac{\sqrt{3}}{2}({T}_{x}{k}_{\{y}{B}_{8{\rm{v}}}^{+}{k}_{z\}}+{\rm{c}}.{\rm{p}}.)\\  &  & +\frac{\sqrt{3}}{2}[({T}_{xx}-{T}_{yy})\,(\frac{2}{3}{k}_{z}{B}_{8{\rm{v}}}^{-}{k}_{z}-\frac{1}{3}{k}_{x}{B}_{8{\rm{v}}}^{-}{k}_{x}-\frac{1}{3}{k}_{y}{B}_{8{\rm{v}}}^{-}{k}_{y})\\  &  & -{T}_{zz}({k}_{x}{B}_{8{\rm{v}}}^{-}{k}_{x}-{k}_{y}{B}_{8{\rm{v}}}^{-}{k}_{y})],\end{array}$$

2e $${H}_{{\rm{6c7v}}}=-\frac{1}{\sqrt{3}}{\boldsymbol{\sigma }}\cdot \tilde{{\boldsymbol{k}}}P-\frac{i}{2\sqrt{3}}({\sigma }_{x}k{}_{[y}B_{7{\rm{v}}}{k}_{z]}+{\rm{c}}.{\rm{p}}.),$$

2f $$\begin{array}{ccc}{H}_{8{\rm{v}}7{\rm{v}}} & = & -\frac{{\hslash }^{2}}{2{m}_{0}}\{\,-6({T}_{xx}^{\dagger }{k}_{x}{\gamma {\rm{^{\prime} }}}_{2}{k}_{x}+{\rm{c}}.{\rm{p}}.)-6({T}_{xy}^{\dagger }k{}_{\{x}{\gamma }_{3{\rm{^{\prime} }}}{k}_{z\}}+{\rm{c}}.{\rm{p}}.)\}\\  &  & -i\frac{\sqrt{3}}{2}({T}_{yz}^{\dagger }\{{C}_{k},{k}_{x}\}+{\rm{c}}.{\rm{p}}.)\\  &  & -3{b}_{{\rm{v}}}({T}_{xx}^{\dagger }{\eta }_{xx}+{\rm{c}}.{\rm{p}}.)-\sqrt{3}{d}_{{\rm{v}}}(2{T}_{xy}^{\dagger }{\eta }_{xy}+{\rm{c}}.{\rm{p}}.)\\  &  & -i\frac{3{\hslash }^{2}}{2{m}_{0}}[k{}_{[x}\kappa {\rm{^{\prime} }}{k}_{y]}{T}_{z}^{\dagger }+{\rm{c}}.{\rm{p}}.]-3({\mu }_{B}{B}_{z}{T}_{z}^{\dagger }+{\rm{c}}.{\rm{p}}.).\end{array}$$

Here $\{{{\mathscr{O}}}_{1},{{\mathscr{O}}}_{2}\}={{\mathscr{O}}}_{1}\,{{\mathscr{O}}}_{2}+{{\mathscr{O}}}_{2}\,{{\mathscr{O}}}_{1}$, ${k}_{\{i}{\mathscr{O}}{k}_{j\}}={k}_{i}{\mathscr{O}}{k}_{j}+{k}_{j}{\mathscr{O}}{k}_{i}$, ${k}_{[i}{{\mathscr{O}}k}_{j]}={k}_{i}{\mathscr{O}}{k}_{j}-{k}_{j}{\mathscr{O}}{k}_{i}$ for any operators ${\mathscr{O}}$, ${{\mathscr{O}}}_{1}$, ${{\mathscr{O}}}_{2}$; *E*_c_ and *E*_v_ are the cb and vb edges, respectively (*E*_0_ = *E*_c_ − *E*_v_ is the fundamental band gap in a bulk crystal); *η* is the strain tensor corresponding to the static deformation due to the lattice mismatch; ${\boldsymbol{k}}=-i\nabla +e{\boldsymbol{A}}/\hslash$, where ***A*** is the vector potential of the magnetic field ***B***; $\tilde{{\boldsymbol{k}}}={\boldsymbol{k}}({\mathbb{I}}-\eta )$; *V*_p_ is the piezoelectric potential; *m*_0_ is the free electron mass; ${A}_{c}^{\prime}$, *g*′ and *κ*′ are given by^3^

3 $${A{\rm{^{\prime} }}}_{c}\equiv \frac{{m}_{0}}{m{\rm{^{\prime} }}}=\frac{{m}_{0}}{{m}^{\ast }}-\frac{2}{3}\frac{{E}_{P}}{{E}_{0}}-\frac{1}{3}\frac{{E}_{P}}{{E}_{0}+{{\rm{\Delta }}}_{0}},\,g{\rm{^{\prime} }}=2,\,\kappa {\rm{^{\prime} }}=-\frac{1}{3}({\gamma {\rm{^{\prime} }}}_{1}-2{\gamma {\rm{^{\prime} }}}_{2}-3{\gamma {\rm{^{\prime} }}}_{3}+2);$$

*P* = *ħ*(*E*_*P*_/2*m*_0_)^1/2^ (see below for the definition of *E*_*P*_); $${\gamma {\rm{^{\prime} }}}_{i}$$ and *κ*′ are the Luttinger parameters with removed contributions from the Γ_6_ cb, $${\gamma {\rm{^{\prime} }}}_{1}={\gamma }_{1}-{E}_{P}/(3{E}_{0}+{{\rm{\Delta }}}_{0})$$, $${\gamma }_{\mathrm{2,3}}^{\prime} ={\gamma }_{\mathrm{2,3}}-{E}_{P}\mathrm{/(6}{E}_{0}+2{{\rm{\Delta }}}_{0})$$, *μ*_*B*_ is the Bohr magneton; *q* is another parameter of the Luttinger Hamiltonian; $${B}_{7{\rm{v}}}=(P{\rm{^{\prime} }}Q/i)\,[1/({E}_{0}-{E{\rm{^{\prime} }}}_{0}-{{\rm{\Delta }}{\rm{^{\prime} }}}_{0})-1/({{\rm{\Delta }}}_{0}+{E{\rm{^{\prime} }}}_{0}+{{\rm{\Delta }}{\rm{^{\prime} }}}_{0})]$$, $${B}_{8{\rm{v}}}^{\pm }=(P^{\prime} Q\mathrm{/2}i)\,[\pm \mathrm{1/(}{E}_{0}-{E}_{0}^{\prime} -{{\rm{\Delta }}}_{0}^{\prime} )\mp \mathrm{1/(}{E}_{0}^{\prime} $$ + $${{\rm{\Delta }}}_{0}^{\prime} )+\mathrm{1/(}{E}_{0}-{E}_{0}^{\prime} )-\mathrm{1/}{E}_{0}^{\prime} ]$$, where *P*′ and *Q* are couplings to higher conduction bands; *σ*_*i*_ are Pauli matrices; *J*_*i*_ are matrices of the *j* = 3/2 representation of angular momentum; *T*_*i*_ are matrix representations of a vector operator between *j* = 1/2 and *j* = 3/2 states, i.e., $${T}_{x}=({T}_{-1}^{\mathrm{(1)}}-{T}_{+1}^{\mathrm{(1)}})/\sqrt{2}$$, $${T}_{y}=-({T}_{-1}^{\mathrm{(1)}}+{T}_{+1}^{\mathrm{(1)}})/\sqrt{2}$$, $${T}_{z}={T}_{0}^{\mathrm{(1)}}$$, with the matrix elements of the spherical components $${T}_{q}^{\mathrm{(1)}}$$ given in terms of the Clebsch-Gordan coefficients 〈*j*_1_*j*_2_; *m*_1_*m*_2_|*jm*〉 by the Wigner-Eckart theorem, $$\langle m|{T}_{q}^{\mathrm{(1)}}|m^{\prime} \rangle \,=\,$$$$-\sqrt{\mathrm{2/3}}\langle \mathrm{3/2},m^{\prime} ;1,q\mathrm{|1/2},m\rangle $$, for *m* = ±1/2, *m*′ = −3/2, …, 3/2; and *T*_*ij*_ = *T*_*i*_*J*_*j*_ + *T*_*j*_*J*_*i*_. The system is placed in an axial magnetic field. In numerical calculations we use gauge-invariant discretization scheme^13^ for the covariant derivative.

The material parameters used in our ***k*** · ***p*** calculations are given in Table 1. In order to avoid ${A}_{c}^{\prime}  < 0$, which would break the ellipticity condition, we rescale *E*_*P*_ to obtain ${A}_{c}^{\prime} =1$, which gives^27^ *E*_*P*_ = (*m*_0_/m* − 1)*E*_0_(*E*_0_ + Δ_0_)/(*E*_0_ + 2Δ_0_/3). Due to inconsistency of the reported values^3,28^, we calculate *q* using the perturbative formula^29^ $q=\mathrm{(2/9)}{E}_{Q}\mathrm{[1/}{E}_{0}^{\prime} -\mathrm{1/(}{E}_{0}^{\prime} +{\Delta }_{0}^{\prime} )]$, where *E*_*Q*_, ${E}_{0}^{\prime}$ and ${\Delta }_{0}^{\prime}$ are 14 band ***k*** · ***p*** parameters^3^. We account for the strain within a continuous elasticity approach^30^. Piezoelectric field in the system is calculated up to the second order in the polarization^31^ with the parameters taken from ref.^32^.

**Table 1:** Material parameters used in the calculations^3,46^.

	GaAs	InAs	Interpolation for In_x_Ga_1−x_As
*E*_v_	0.0 eV	0.21 eV	linear
*E*_0_	1.519 eV	0.417 eV	0.417*x* + 1.519(1 − *x*) − 0.477*x*(1 − *x*)
${E}_{{\rm{0}}}^{\prime}$	4.488 eV	4.390 eV	linear
*E*_Q_	17.535 eV	18.255 eV	linear
m*	0.0665*m*_0_	0.0229*m*_0_	[0.0229*x* + 0.0665(1 − *x*) − 0.0091*x*(1 − *x*)]*m*_0_
Δ	0.341 eV	0.39 eV	0.39*x* + 0.341(1 − *x*) − 0.15*x*(1 − *x*)
${{\rm{\Delta }}^{\prime} }_{0}$	0.171 eV	0.25 eV	linear
*a*_c_	−7.17 eV	−5.08 eV	−5.08*x* − 7.17(1 − *x*) − 2.61*x*(1 − *x*)
*a*_v_	1.16 eV	1.00 eV	linear
*b*_v_	−2.0 eV	−1.8 eV	linear
*d*_v_	−4.8 eV	−3.6 eV	linear
*γ*_1_	6.98	20.0	1/[(1 − *x*)/6.98 + *x*/20.0]
*γ*_2_	2.06	8.5	1/[(1 − *x*)/8.5 + *x*/2.06]
*γ*_3_	2.93	9.2	1/[(1 − *x*)/9.2 + *x*/2.93]
*P*′	4.780*i*	0.873*i*	linear
*Q*	8.165	8.331	linear

## Derivation of the effective mass Hamiltonian

The essence of the method^24,33^ is to perturbatively decouple the group of states of interest from all the other states by using a canonical transformation *T* = *e*^*S*^, with an anti-hermitian operator *S*, in order to obtain a transformed Hamiltonian $\tilde{H}=TH{T}^{\dagger }$, in which the inter-band terms (treated as a perturbation) are approximately eliminated. We will use a modified version of the van Vleck quasi-degenerate perturbation theory^34^ as presented in ref.^35^. The Hamiltonian is split into its block-diagonal and block-off-diagonal parts (coupling states within a single group of states and between the two groups of states, respectively), *H* = *H*^(d)^ + *H*^(od)^. The operator *S* is required to have null matrix elements within the groups (that is, it has to be block-off-diagonal). The group of states of interest here are the cb states. Since we start from the 8-band ***k*** · ***p*** theory, the other group of states are vb states.

We define the superoperator $\hat{S}$ representing the adjoint action of *S* on the algebra of operators: $\hat{S}{\mathscr{O}}=[{\mathscr{O}},S]$, ${\hat{S}}^{2}{\mathscr{O}}=[[{\mathscr{O}},S],S]$, etc. for any operator ${\mathscr{O}}$. Functions of $\hat{S}$ are defined via their power series expansion. Then, from the Campbell-Baker-Hausdorff expansion, $\tilde{H}={e}^{S}H{e}^{-S}={e}^{\hat{S}}H=\,\cosh \,\hat{S}H+\,\sinh \,\hat{S}H$. Even functions of $\hat{S}$ transform block-diagonal operators into block-diagonal operators and block-off-diagonal operators into block-off-diagonal operators, and the opposite holds for odd functions of $\hat{S}$. Therefore, the block-diagonal and block-off-diagonal parts of $\tilde{H}$ are

4 $${\tilde{H}}^{({\rm{d}})}=\,\cosh \,\hat{S}{H}^{({\rm{d}})}+\,\sinh \,\hat{S}{H}^{({\rm{od}})},\,{\tilde{H}}^{({\rm{od}})}=\,\sinh \,\hat{S}{H}^{({\rm{d}})}+\,\cosh \,\hat{S}{H}^{({\rm{od}})}.$$

We require that the resulting Hamiltonian is block-diagonal, hence ${\tilde{H}}^{({\rm{od}})}=0$. Inverting Eq. (4) then yields

5 $${H}^{({\rm{od}})}=-\sinh \,\hat{S}{\tilde{H}}^{({\rm{d}})}\mathrm{.}$$

In our case *H*^(od)^ consists of *H*_6c8v_, *H*_6c7v_ [Eqs (2d) and (2e)] and their hermitian conjugates, $${H}^{({\rm{od}})}={H}_{{\rm{cv}}}+{\rm{h}}.{\rm{c}}.=$$$${H}_{{\rm{6c8v}}}\oplus {H}_{{\rm{6c7v}}}+{\rm{h}}.{\rm{c}}.$$, hence it contains terms linear and quadratic in ***k***. Since $${\tilde{H}}^{({\rm{d}})}$$ contains *k*-independent terms, *S* must be *O*(*k*). According to the first of Eq. (4), the leading order terms in *S* yield quadratic corrections to $${\tilde{H}}^{({\rm{d}})}$$. We therefore take into account only the linear term in Eq. (5) and write

6 $${H}^{({\rm{od}})}=-\hat{S}{\tilde{H}}^{({\rm{d}})}=[S,{\tilde{H}}^{({\rm{d}})}\mathrm{].}$$

The neglected corrections are *O*(*k*^3^) and would lead to quartic terms in ${\tilde{H}}^{({\rm{d}})}$, which is beyond the effective mass approximation, even with spin-orbit terms. Quantitatively, the linear truncation according to Eq. (6) amounts to neglecting corrections of relative magnitude Δ*E*/*E*_0_, where Δ*E* is the interband energy separation due to confinement. Taking Δ*E* as the excitation energy in the direction of strongest confinement (~200 meV) one gets a rough estimate of 20% for the error due to truncation.

The remaining, block-diagonal part of the transformed Hamiltonian is now written as ${\tilde{H}}^{({\rm{d}})}={H}_{0}+{\tilde{H}}_{{\rm{c}}}^{\prime} \oplus {\tilde{H}}_{{\rm{v}}}^{\prime}$, where *H*_0_ is assumed to be diagonal in a certain basis |*α*, *i*〉 (where *α* denotes a group of states and *i* are individual states within these groups), and is selected in such a way that the remaining parts are in some sense small. ${\tilde{H}}_{{\rm{c}}}^{\prime}$ and ${\tilde{H}}_{{\rm{v}}}^{\prime} \,$ denote the cb and vb blocks of $\tilde{H}$, respectively, with the corresponding parts of *H*_0_ subtracted. In the problem at hand, we choose for *H*_0_ a diagonal Hamiltonian which is constant and proportional to unity within each of the 6c, 8v and 7v bands and approximately represents the band edges in a strained QD. We will denote the respective energy values by ${\overline{E}}_{\alpha }$, *α* = 6c, 8v, 7v. One can understand them as the average band edges corrected by hydrostatic strain (while the splitting between heavy and light holes within the 8v band is not included). The operator *S* is written as *S* = *S*_cv_ + h.c., where *S*_cv_ denotes one of the two off-diagonal blocks (a 2 × 6 matrix in the standard ***k*** · ***p*** matrix notation). The effective band gaps are denoted by ${\overline{E}}_{{\rm{6c}}}-{\overline{E}}_{{\rm{8v}}}={E}_{{\rm{g}}}$ and ${\overline{E}}_{{\rm{6c}}}-{\overline{E}}_{{\rm{7v}}}={E}_{{\rm{g}}}+{{\rm{\Delta }}}_{{\rm{SO}}}$. Finally we define a diagonal operator $\hat{{\rm{\Delta }}}={\rm{diag}}({E}_{{\rm{g}}},{E}_{{\rm{g}}},{E}_{{\rm{g}}},{E}_{{\rm{g}}},{E}_{{\rm{g}}}+{{\rm{\Delta }}}_{{\rm{SO}}},{E}_{{\rm{g}}}+{{\rm{\Delta }}}_{{\rm{SO}}})$, where the entries correspond to the 6 valence bands of the 8-band ***k*** · ***p*** model. Then, from Eq. (6), one finds

7 $${\tilde{H}}_{{\rm{c}}}^{\prime} {S}_{{\rm{cv}}}-{S}_{{\rm{cv}}}({\tilde{H}}_{{\rm{v}}}^{\prime} -\hat{{\rm{\Delta }}})=-{H}_{{\rm{cv}}}.$$

Note that the arbitrariness of choosing the diagonal Hamiltonian *H*_0_ is removed here, as the subtracted energies (the operator $\hat{{\rm{\Delta }}}$) are added back to the remaining part of the Hamiltonian.

Equation (7) has a structure of a Sylvester equation but the operators appearing here are not finite-dimensional matrices. One can treat this equation as a matrix one, in the sense of the block notation over the subbands, but then the problem of non-commutativity of the matrix elements appears (due to non-commutativity of ***k*** with position-dependent quantities), precluding the application of standard algebraic methods for solving this equation. In order to overcome this difficulty, we expand the operators *S*_cv_ and *H*_cv_ in powers of ***k***,

$${S}_{{\rm{cv}}}={X}^{\mathrm{(0)}}+\sum _{j}\,{k}_{j}{X}_{j}^{\mathrm{(1)}}+\sum _{jl}\,{k}_{j}{X}_{jl}^{\mathrm{(2)}}{k}_{l},\,{H}_{{\rm{cv}}}=-\sum _{j}\,{k}_{j}{C}_{j}^{\mathrm{(1)}}-\sum _{jl}\,{k}_{j}{C}_{jl}^{\mathrm{(2)}}{k}_{l},$$

where the coefficients *C*^(1)^ and *C*^(2)^ are defined by comparison with the explicit form of Eqs (2d) and (2e). Then, upon rearrangement of terms one gets from Eq. (7) in the subsequent (formal) orders in ***k***

8a $${\tilde{H}}_{{\rm{c}}}^{\prime} {X}_{jl}^{\mathrm{(2)}}-{X}_{jl}^{\mathrm{(2)}}({\tilde{H}}_{{\rm{v}}}^{\prime} -\hat{{\rm{\Delta }}})={C}_{jl}^{\mathrm{(2)}},$$

8b $${\tilde{H}}_{{\rm{c}}}^{\prime} {X}_{j}^{\mathrm{(1)}}-{X}_{j}^{\mathrm{(1)}}({\tilde{H}}_{{\rm{v}}}^{\prime} -\hat{{\rm{\Delta }}})={C}_{j}^{\mathrm{(1)}}+[{\tilde{H}}_{{\rm{c}}}^{\prime} ,{k}_{l}]{X}_{lj}^{\mathrm{(2)}}+{X}_{jl}^{\mathrm{(2)}}[{k}_{l},{\tilde{H}}_{{\rm{v}}}^{\prime} -\hat{{\rm{\Delta }}}],$$

8c $${\mathop{H}\limits^{ \sim }{\rm{^{\prime} }}}_{{\rm{c}}}{X}^{(0)}-{X}^{(0)}({\mathop{H}\limits^{ \sim }{\rm{^{\prime} }}}_{{\rm{v}}}-\hat{{\rm{\Delta }}})=-[{\mathop{H}\limits^{ \sim }{\rm{^{\prime} }}}_{{\rm{c}}},{k}_{j}]{X}_{j}^{(1)}-[[{\mathop{H}\limits^{ \sim }{\rm{^{\prime} }}}_{{\rm{c}}},{k}_{j}]{X}_{jl}^{(2)},{k}_{l}].$$

The non-commutativity problem persists since ${\tilde{H}}_{{\rm{c}}}^{\prime}$ and ${\tilde{H}}_{{\rm{v}}}^{\prime}$ contain *k*-dependent terms, while *X*^(*n*)^ are position-dependent. Returning to Eq. (6) one can see that the terms of ${\tilde{H}}^{({\rm{d}})}$ linear and quadratic in *k* generate corrections to *S* on the order of *k*^2^ and *k*^3^, respectively. According to Eq. (4), these corrections generate terms *O*(*k*^3^) and *O*(*k*^4^) in the effective mass Hamiltonian for the cb. The latter are beyond the usual effective mass approximation, while the former correspond to spin-orbit terms but (by a simple perturbation argument) appear with a coefficient $\sim {P}^{2}{C}_{k}/{E}_{{\rm{g}}}^{2}\sim 0.6$ nm^3^ · meV (using InAs parameters), which is two orders of magnitude smaller than the Dresselhaus coefficient for InAs, *α*_D_ = 27 nm^3^ · meV. It appears, therefore, that the kinetic part of ${\mathop{H}\limits^{ \sim }{\rm{^{\prime} }}}_{{\rm{v}}}$ can be discarded in the derivation of a usual effective mass equation in the parabolic band approximation, that is, one with *O*(*k*^2^) kinetic terms and the relevant *O*(*k*^3^) spin-orbit corrections. However, as we will see below, including non-parabolicity effects at this point improves accuracy of the modeling of a self-assembled QD. Therefore, it seems reasonable to keep the kinetic part of ${\tilde{H}}_{{\rm{c}}}^{\prime}$ and ${\tilde{H}}_{{\rm{v}}}^{\prime}$. In order to obtain a solvable system of equations, we therefore propose to self-consistently replace the ***k***-dependent terms by their averages in the eigenstate of interest.

In our derivation, Eqs (8a)–(8c) depend on the blocks of the transformed Hamiltonian $\tilde{H}$ rather than on the initial Hamiltonian *H* hence, together with Eq. (4), they form a system that cannot be solved in a closed form. Clearly, in the leading order one could replace ${\tilde{H}}_{{\rm{c}}}^{\prime}$ and ${\tilde{H}}_{{\rm{v}}}^{\prime}$ by the original blocks ${\mathop{H}\limits^{ \sim }{\rm{^{\prime} }}}_{{\rm{c}}}$ and ${\mathop{H}\limits^{ \sim }{\rm{^{\prime} }}}_{{\rm{v}}}$ (the correction is *O*(*k*^2^), yielding corrections that are formally *O*(*k*^4^) in the resulting cb Hamiltonian). However, as we will see, including at least some corrections to these blocks improves the accuracy of the equation. It is known that the major corrections to the cb and vb Hamiltonians resulting from the decoupling procedure are the renormalization of the cb electron mass and of the Luttinger parameters, respectively. Therefore, we propose to take into account these strong effects only and to use, in place of ${\tilde{H}}_{{\rm{c}}}^{\prime}$ and ${\tilde{H}}_{{\rm{v}}}^{\prime}$, the cb and vb blocks of the original 8-band Hamiltonian but with the position-dependent renormalized parameters,

9 $$\frac{{m}_{0}}{\tilde{m}}=\frac{{m}_{0}}{m^{\prime} }+\frac{2}{3}\frac{{E}_{P}}{{E}_{g}^{\prime} }+\frac{1}{3}\frac{{E}_{P}}{{E}_{g}^{\prime} +{{\rm{\Delta }}}_{0}},\,{\tilde{\gamma }}_{1}={\gamma ^{\prime} }_{1}+\frac{{E}_{P}}{3{E}_{g}^{\prime} +{{\rm{\Delta }}}_{0}},\,{\tilde{\gamma }}_{\mathrm{2,3}}={\gamma }_{\mathrm{2,3}}^{\prime} +\frac{1}{2}\frac{{E}_{P}}{3{E}_{g}^{\prime} +{{\rm{\Delta }}}_{0}},$$

where ${E{\rm{^{\prime} }}}_{{\rm{g}}}={E}_{0}+({a}_{{\rm{c}}}-{a}_{{\rm{v}}})\,{\rm{T}}{\rm{r}}\eta$ is the local band gap, including the hydrostatic strain-induced shift. Note that these band-decoupling corrections are *O*(*k*^2^), so the distinction between ${\tilde{H}}_{c,v}^{\prime}$ and ${H}_{c,v}^{\prime}$ is only important if the *k*-dependent terms are included self-consistently, as proposed above.

In the approximations proposed here, Eqs (8a)–(8c) are a system of usual Sylvester equations that can be solved iteratively. A solution can be obtained in a closed, analytical form using the general method of ref.^36^. However, the form of the solution simplifies considerably if one discards the contribution of the spin-dependent part of ${\tilde{H}}_{{\rm{c}}}^{\prime}$ to the operator *S* (these terms remain included to the leading order in *H*_6c6c_ that is part of *H*^(d)^, see Eqs (2a) and (4)). These terms are very small compared to any other energy scales in the problem, hence their contribution to *S* is negligible. Within this approximation, one has ${\tilde{H}}_{{\rm{c}}}^{\prime} ={\chi }_{{\rm{c}}}^{\prime} {{\mathbb{I}}}_{2\times 2}$, where ${\chi }_{{\rm{c}}}^{\prime}$ is a scalar function of position and ${{\mathbb{I}}}_{2\times 2}$ is a 2 × 2 unit matrix. Then, the solution to the above system of equations can be obtained trivially. Denoting ${\mathscr{D}}=\hat{{\rm{\Delta }}}+{\chi }_{{\rm{c}}}^{\prime} {{\mathbb{I}}}_{6\times 6}-{\tilde{H}}_{{\rm{v}}}^{\prime}$ one has

$$\begin{array}{rcl}{X}_{jl}^{\mathrm{(2)}} & = & {C}_{jl}^{\mathrm{(2)}}{{\mathscr{D}}}^{-1},\\ {X}_{j}^{\mathrm{(1)}} & = & {C}_{j}^{\mathrm{(1)}}{{\mathscr{D}}}^{-1}+{C}_{jl}^{\mathrm{(2)}}[{k}_{l},{{\mathscr{D}}}^{-1}],\\ {X}^{\mathrm{(0)}} & = & {C}_{j}^{\mathrm{(1)}}[{k}_{j},{\chi }_{{\rm{c}}}^{\prime} ]{{\mathscr{D}}}^{-2}-[{k}_{l},[{k}_{j},{\chi }_{{\rm{c}}}^{\prime} ]{C}_{jl}^{\mathrm{(2)}}]\,{{\mathscr{D}}}^{-2}\mathrm{.}\end{array}$$

From Eq. (4), with the condition ${\mathop{H}\limits^{ \sim }}^{({\rm{o}}{\rm{d}})}=0$, one finds^35^ ${\mathop{H}\limits^{ \sim }}^{({\rm{d}})}={H}^{({\rm{d}})}+\,\tanh (\hat{S}/2){H}^{({\rm{o}}{\rm{d}})}$ or, in the leading order, ${\mathop{H}\limits^{ \sim }}^{({\rm{d}})}\approx {H}^{({\rm{d}})}+(1/2)\,[{H}^{({\rm{o}}{\rm{d}})},S]$. Then, the correction to the cb Hamiltonian up to the order *k*^3^ can be decomposed into two parts. The first one, which we will denote by ${\tilde{H}}^{(2)}$, is formally quadratic in ***k*** and is proportional to ${C}_{j}^{(1)}{C}_{l}^{(1)}$ in our notation. This contribution yields corrections to the electron effective mass and Landé factor. The second part, denoted ${\tilde{H}}^{(3)}$, is of third order in ***k*** and contains terms proportional to ${C}_{jl}^{(2)}{C}_{n}^{(1)}$. It includes the Dresselhaus spin-orbit term. Thus, the resulting transformed Hamiltonian can be written as $\tilde{H}={H}_{{\rm{c}}}+{\tilde{H}}^{(2)}+{\tilde{H}}^{(3)}$. From Eq. (4) in the linear approximation one finds

10 $${\tilde{H}}^{\mathrm{(2)}}=\sum _{jl}\,{k}_{j}{C}_{j}^{\mathrm{(1)}}{{\mathscr{D}}}^{-1}{C}_{l}^{\mathrm{(1)}\dagger }{k}_{l}+\frac{1}{2}\,\sum _{jl}\,([{k}_{j},{\chi }_{{\rm{c}}}^{\prime} ]{C}_{j}^{\mathrm{(1)}}{{\mathscr{D}}}^{-2}{C}_{l}^{\mathrm{(1)}\dagger }{k}_{l}+{\rm{h}}.{\rm{c}}.)$$

and

11 $$\begin{array}{rcl}{\tilde{H}}^{\mathrm{(3)}} & = & \sum _{jln}\,{k}_{j}{C}_{jl}^{\mathrm{(2)}}{k}_{l}{{\mathscr{D}}}^{-1}{C}_{n}^{\mathrm{(1)}\dagger }{k}_{l}+\frac{1}{2}\,\sum _{jln}\,\{-[{k}_{l},[{k}_{j},{\chi }_{{\rm{c}}}^{\prime} ]{C}_{jl}^{\mathrm{(2)}}]\,{{\mathscr{D}}}^{-2}{C}_{n}^{\mathrm{(1)}\dagger }{k}_{n}\\  &  & +\,{k}_{l}{C}_{jl}^{\mathrm{(2)}}{k}_{j}{{\mathscr{D}}}^{-2}{C}_{n}^{\mathrm{(1)}\dagger }[{k}_{n},{\chi }_{{\rm{c}}}^{\prime} ]+{\rm{h}}.{\rm{c}}.\}.\end{array}$$

The first term in Eq. (11) leads to the usual Dresselhaus spin-orbit coupling. The other terms are linear or quadratic in *k* and yield a small correction to the kinetic and Zeeman terms of the effective mass Hamiltonian.

## Interpretation, Verification and Discussion

In this Section, we first present various approximations to the effective mass Hamiltonian and relate the equations resulting from some of these approximations to the common form of the effective mass Hamiltonian, written in terms of the effective mass tensor and the *g*-factor given by the Roth formula. Next we define a series of approximation for which we present quantitative comparison of the predictions from the effective mass theory with the results from the 8-band ***k*** · ***p*** Hamiltonian.

### Interpretation of the effective mass equation

We restrict the discussion to the quadratic term [Eq. (10)] that determines the fundamental properties of the energy spectrum.

In order to simplify notation, we define the 2 × 6 matrices ${{\mathscr{T}}}_{i}=\sqrt{3}{T}_{i}\oplus (-\mathrm{1/}\sqrt{3}){\sigma }_{i}$, where the two components of the direct sum correspond to the *j* = 3/2 (hh and lh) and *j* = 1/2 (spin-orbit split-off) subbands of the vb. Then, by direct inspection of Eqs (2d) and (2e) one finds ${C}_{j}^{\mathrm{(1)}}=-P{\tilde{{\mathscr{T}}}}_{j}$, where ${\tilde{{\mathscr{T}}}}_{j}={\sum }_{n}\,({\delta }_{jn}-{\eta }_{jn}){{\mathscr{T}}}_{n}$. The second-order correction to the effective mass Hamiltonian is then

12 $${\tilde{H}}^{\mathrm{(2)}}=\sum _{jl}\,{k}_{j}P{\tilde{{\mathscr{T}}}}_{j}{{\mathscr{D}}}^{-1}{\tilde{{\mathscr{T}}}}_{l}^{\dagger }P{k}_{l}+\frac{1}{2}\,\sum _{jl}\,(P{\tilde{{\mathscr{T}}}}_{j}{{\mathscr{D}}}^{-1}[{k}_{j},{\chi }_{{\rm{c}}}^{\prime} ]\,{{\mathscr{D}}}^{-1}{\tilde{{\mathscr{T}}}}_{l}^{\dagger }P{k}_{l}+{\rm{h}}.{\rm{c}}.),$$

where we use the fact that $[{k}_{j},{\chi }_{{\rm{c}}}^{\prime} ]$ is a number and commutes with the matrices ${\tilde{{\mathscr{T}}}}_{i}$ and ${\mathscr{D}}$.

It might seem that the above Hamiltonian confirms the correctness of the particular (BenDaniel-Duke^37^) ordering of the operators in the kinetic term. This is not the case: a simple manipulation of the terms in Eq. (12) under assumption *P* = const allows one to rewrite the second-order correction in the equivalent form

13 $${\mathop{H}\limits^{ \sim }}^{(2)}=\frac{1}{2}\,\sum _{jl}\,\{{k}_{j}{k}_{l},P{\mathop{{\mathscr{T}}}\limits^{ \sim }}_{j}{{\mathscr{D}}}^{-1}{\mathop{{\mathscr{T}}}\limits^{ \sim }}_{l}^{\dagger }P\}+\frac{1}{2}\,\sum _{jl}\,(P{\mathop{{\mathscr{T}}}\limits^{ \sim }}_{j}{{\mathscr{D}}}^{-1}[{k}_{j},{H{\rm{^{\prime} }}}_{{\rm{v}}}]\,{{\mathscr{D}}}^{-1}{\mathop{{\mathscr{T}}}\limits^{ \sim }}_{l}^{\dagger }P{k}_{l}+{\rm{h}}.{\rm{c}}.),$$

where a different (Gora-Williams-Bastard^38–40^) ordering appears in the kinetic term. Interestingly, the correcting terms in Eqs (12) and (13) involve only spatial derivatives of cb and vb parameters, respectively. It is hence clear that the two orderings are, in a sense, dual and each of them corresponds to neglecting terms in the Hamiltonian, related to spatial modulation of either the conduction or valence band. As we will see explicitly below, these additional terms are essential to correctly reproduce the Rashba spin-orbit interaction.

Further analytical insight into the somewhat unusual form of our effective mass Hamiltonian $\tilde{H}$ is hindered by the need to invert the “band gap operator” ${\mathscr{D}}$, which produces rather intransparent and intractable formulas (and, in practice, is performed numerically). One might expect that neglecting the off-diagonal elements of ${\mathscr{D}}$, from which this difficulty stems, is a good approximation, since these elements are rather small compared to the band gap. The operator ${\mathscr{D}}$ in this approximation will be denoted by $\tilde{{\mathscr{D}}}$. Since it is diagonal its inverse powers are found trivially. Let us denote its diagonal elements by (*E*_hh_, *E*_lh_, *E*_lh_, *E*_hh_, *E*_so_, *E*_so_). They can be interpreted as the offset of the local edges of the three valence subbands with respect to the cb edge at a given point in space. We split the ${{\mathscr{T}}}_{j}{\tilde{{\mathscr{D}}}}^{-n}{{\mathscr{T}}}_{l}^{\dagger }$ matrix into a symmetric and asymmetric part,

14 $${({\tilde{{\mathscr{T}}}}_{j}{\tilde{{\mathscr{D}}}}^{-n}{\tilde{{\mathscr{T}}}}_{l}^{\dagger })}_{{\rm{s}},{\rm{as}}}=\frac{1}{2}\,({\tilde{{\mathscr{T}}}}_{j}{\tilde{{\mathscr{D}}}}^{-n}{\tilde{{\mathscr{T}}}}_{l}^{\dagger }\pm {\tilde{{\mathscr{T}}}}_{l}{\tilde{{\mathscr{D}}}}^{-n}{\tilde{{\mathscr{T}}}}_{j}^{\dagger })\mathrm{.}$$

Using the explicit forms of the matrices ${{\mathscr{T}}}_{j}$ one finds up to the linear order in *η*

$${({\tilde{{\mathscr{T}}}}_{j}{\tilde{{\mathscr{D}}}}^{-n}{\tilde{{\mathscr{T}}}}_{l}^{\dagger })}_{{\rm{s}}}=\frac{{\hslash }^{2}}{2{m}_{0}{P}^{2}}({\delta }_{jl}-2{\eta }_{jl})\frac{{F}_{j}^{(n)}+{F}_{l}^{(n)}}{2}{{\mathbb{I}}}_{2\times 2},$$

where ${F}_{x}^{(n)}={F}_{y}^{(n)}={E}_{{\rm{P}}}\mathrm{(2/}{E}_{{\rm{so}}}^{n}+\mathrm{1/}{E}_{{\rm{lh}}}^{n}+3{E}_{{\rm{hh}}}^{n}\mathrm{)/6}$, ${F}_{z}^{(n)}={E}_{{\rm{P}}}\mathrm{(1/}{E}_{{\rm{so}}}^{n}+\mathrm{2/}{E}_{{\rm{lh}}}^{n}\mathrm{)/3}$. Thus, the symmetric part is spin-independent and contributes to the kinetic part of the effective mass Hamiltonian in the well-known way via the electron effective mass tensor; indeed, the first term in Eq. (12) combined with the kinetic term in *H*_6c6c_ [Eq. (2a)] is

15 $${\tilde{H}}^{({\rm{s}}\mathrm{,1)}}=\sum _{j}\,\frac{{\hslash }^{2}{k}_{j}^{2}}{2{m}_{j}^{\ast }},\quad {\rm{where}}\quad \frac{{m}_{0}}{{m}_{j}^{\ast }}=\frac{{m}_{0}}{m^{\prime} }+{F}_{j}^{\mathrm{(1)}}.$$

The second term yields

16 $${\tilde{H}}^{({\rm{s}}\mathrm{,2)}}=-\frac{{\hslash }^{2}}{4{m}_{0}}\,\sum _{jl}\,[{k}_{l},[{k}_{j},{\chi }_{{\rm{c}}}^{\prime} ]\,(\frac{1}{2}{\delta }_{jl}-{\eta }_{jl})\,({F}_{j}^{\mathrm{(2)}}+{F}_{l}^{\mathrm{(2)}})].$$

Thus, the symmetric component of the first term in Eq. (12) yields the usual kinetic energy of the effective mass theory (Eq. (15)) in the commonly used ordering “*k*(m*)^−1^*k*” (BenDaniel-Duke^37^ ordering). This is, however, corrected by the second term (Eq. (16)) and one could as well start from Eq. (13) and arrive at an equivalent Hamiltonian with Gora-Williams-Bastard^38–40^ ordering and with an *H*_v_-dependent correction term instead of Eq. (16).

The asymmetric part of *H*^(2)^ can be interpreted most easily starting form the alternative form of the Hamiltonian given in Eq. (13). The asymmetric contribution to the first term in Eq. (13), combined with the Zeeman term of *H*_6c6c_ [Eq. (2a)] can be written in the form

$${\tilde{H}}^{({\rm{as}},\mathrm{1)}}=\frac{1}{4}\,\sum _{jl}\,\{{k}_{j},[{k}_{l},{{\mathscr{A}}}_{jl}]\},\quad {\rm{where}}\quad {{\mathscr{A}}}_{jl}=\frac{i{\hslash }^{2}g^{\prime} }{4{m}_{0}}\,\sum _{m}\,{\varepsilon }_{jlm}{\sigma }_{m}+P{({\tilde{{\mathscr{T}}}}_{j}{{\mathscr{D}}}^{-1}{\tilde{{\mathscr{T}}}}_{l}^{\dagger })}_{{\rm{as}}}P\mathrm{.}$$

In the diagonal approximation, ${\mathscr{D}}\approx \tilde{{\mathscr{D}}}$, the asymmetric part yields up to order *O*(*η*)

17 $${({\tilde{{\mathscr{T}}}}_{j}{\tilde{{\mathscr{D}}}}^{-n}{\tilde{{\mathscr{T}}}}_{l}^{\dagger })}_{{\rm{as}}}=-\frac{i}{2{E}_{{\rm{P}}}}\,\sum _{m}\,[{\varepsilon }_{jlm}-\sum _{n}\,({\eta }_{jn}{\varepsilon }_{nlm}+{\eta }_{nl}{\varepsilon }_{jnm})]\,\delta {g}_{m}^{(n)}{\sigma }_{m},$$

where

18 $$\delta {g}_{x}^{(n)}=\delta {g}_{y}^{(n)}=\mathrm{(2/3)}{E}_{{\rm{P}}}\mathrm{(1/}{E}_{{\rm{lh}}}^{n}-\mathrm{1/}{E}_{{\rm{so}}}^{n}),\delta {g}_{z}^{(n)}={E}_{{\rm{P}}}[-\mathrm{1/(3}{E}_{{\rm{lh}}}^{n})+\mathrm{1/}{E}_{{\rm{hh}}}^{n}-\mathrm{2/(3}{E}_{{\rm{so}}}^{n})].$$

Using the relation $$[{k}_{j},{k}_{l}]=-\frac{ie}{\hslash }{\varepsilon }_{jlm}{B}_{m^{\prime} }$$, and defining the (diagonal) Landé tensor $${\hat{g}}_{mm^{\prime} }=g^{\prime} {\delta }_{mm^{\prime} }-$$$$[{\delta }_{mm^{\prime} }\mathrm{(1}+{\rm{Tr}}\eta )-{\eta }_{mm^{\prime} }]\,\delta {g}_{m}^{\mathrm{(1)}}$$, one gets the usual Zeeman Hamiltonian

19 $${\tilde{H}}^{({\rm{as}},\mathrm{1)}}\approx \frac{1}{2}{\mu }_{{\rm{B}}}{\boldsymbol{B}}\hat{g}{\boldsymbol{\sigma }}.$$

The form of the Landé tensor $\hat{g}$ appearing in this equation is close to the widely used isotropic Roth-Lax-Zwerdling^41^ formula

20 $${g}^{({\rm{Roth}})}=g^{\prime} -\frac{2{E}_{{\rm{P}}}}{3}(\frac{1}{{E}_{{\rm{g}}}}-\frac{1}{{E}_{{\rm{g}}}+{{\rm{\Delta }}}_{{\rm{SO}}}}).$$

Here, $\hat{g}$ differs from *g*^(Roth)^ by including strain effects as well as anisotropy resulting from the hh-lh splitting in the nanostructure. The Roth-Lax-Zwerdling formula has the advantage that the fundamental band gap *E*_g_, instead of being calculated at some level of approximation to the ***k*** · ***p*** theory, can be taken from experiment^42–44^. While the standard Roth-Lax-Zwerdling formula is linked to the 8-band ***k*** · ***p*** Hamiltonian via a diagonal and parabolic approximations, the full effective mass Hamiltonian accounts also for non-parabolicity corrections and the full structure of the vb.

The asymmetric part of the second term in Eq. (13) is a generalized Rashba term. To see this, assume that the spatial variation of ${H}_{{\rm{v}}}^{\prime}$ results from an external electric field ${\mathcal E}$, hence $[{k}_{j},{H}_{{\rm{v}}}^{\prime} ]=-i\nabla {H}_{{\rm{v}}}^{\prime} =-ie {\mathcal E} {{\mathbb{I}}}_{6\times 6}$. Then, taking only the strain-independent terms in the second term in Eq. (13) and using Eq. (17) one finds in the diagonal approximation

$${H}^{({\rm{as}},{\rm{2}})}=\frac{e{P}^{2}}{4{E}_{{\rm{P}}}}\,\sum _{jlm}\,{ {\mathcal E} }_{j}{\varepsilon }_{jlm}\delta {g}_{m}^{\mathrm{(2)}}{\sigma }_{{\rm{m}}}{k}_{l}+{\rm{h}}.{\rm{c}}.$$

Neglecting the anisotropy due to the lh-hh splitting in Eq. (18) one finds

$${H}^{({\rm{as}},{\rm{2}})}=\frac{e{P}^{2}}{3}(\frac{1}{{E}_{{\rm{g}}}^{2}}-\frac{1}{{({E}_{{\rm{g}}}+{{\rm{\Delta }}}_{{\rm{SO}}})}^{2}})\frac{1}{2} {\mathcal E} \cdot ({\boldsymbol{\sigma }}\times {\boldsymbol{k}})+{\rm{h}}.{\rm{c}}.,$$

which is the standard Rashba spin-orbit term^3^. The full Eq. (13) yields the Rashba term generalized to arbitrary inhomogeneity of the valence band and includes corrections due to inhomogeneous strain. The same effects are accounted for (although not so explicitly) by Eq. (12).

### Quantitative assessment

In the following, we compare the accuracy (with respect to the 8-band ***k*** · ***p*** results) of various approximations to the effective mass equation derived above. Our systematic approach allows us to discuss the possible approximate equations in the uniform framework, according to the level of approximation made to the ${\mathscr{D}}$ operator.

1. As the very first step, obviously inaccurate but included for completeness, we apply the *bulk approximation without strain*, in which the operator ${\mathscr{D}}$ is taken diagonal and without the kinetic (*k*^2^) terms,
  21 $${\mathscr{D}}=\tilde{{\mathscr{D}}}={\rm{diag}}({E}_{{\rm{hh}}},{E}_{{\rm{lh}}},{E}_{{\rm{lh}}},{E}_{{\rm{hh}}},{E}_{{\rm{so}}},{E}_{{\rm{so}}}),$$
  with local bad gaps calculated from unstrained bulk values, *E*_hh_ = *E*_lh_ = *E*_0_, *E*_so_ = *E*_0_ + Δ_0_, where the band edges are interpolated according to the local composition (see Table 1).
2. The first reasonable approximation is to correct the above procedure by accounting for band-edge shifts due to local strain, which is done by including the diagonal strain-dependent matrix elements in *H*_v_ and *H*_c_. Thus, the ${\mathscr{D}}$ operator is still given by Eq. (21) but with *E*_hh_ = *E*_0_ + (*a*_c_ − *a*_v_) Tr*η* + *b*_v_(2*η*_*zz*_ − *η*_*xx*_ − *η*_*yy*_)/2, *E*_lh_ = *E*_0_ + (*a*_c_ − *a*_v_) Tr*η* − *b*_v_(2*η*_*zz*_ − *η*_*xx*_ − *η*_*yy*_)/2, *E*_so_ = *E*_0_ + Δ_0_ + (*a*_c_ − *a*_v_) Tr*η*, where the local value of strain is used and parameters are interpolated according to the local composition. In this way, the major correction to the band gap (which is a crucial factor determining the effective mass and *g*-factor) is included in the model. This will be referred to as *bulk approximation with strain*.
3. The value used for the band gap in the previous approach is still a crude approximation since the actual energy spacing between the states in the conduction and valence bands in a QD are affected by spatial confinement. A common approach, taken in many cases^42–44^ to estimate the electron *g*-factor when interpreting experimental data, is to use the measured value of the fundamental transition energy ${E}_{{\rm{g}}}^{(\exp )}$ as the effective band gap value for a given system. In our numerical study this corresponds to replacing the fundamental band edge offset in the operator ${\mathscr{D}}$ (together with its strain correction) by the splitting between the top vb and bottom cb states obtained from the full 8-band calculation, while using the bulk values of the spin-orbit splitting Δ_0_ and the Kane parameter *P* interpolated according to composition. The ${\mathscr{D}}$ operator is still given by the diagonal form of Eq. (21) but now ${E}_{{\rm{hh}}}={E}_{{\rm{lh}}}={E}_{{\rm{g}}}^{(\exp )}$, ${E}_{{\rm{so}}}={E}_{{\rm{g}}}^{(\exp )}+{{\rm{\Delta }}}_{0}$, with a constant ${E}_{{\rm{g}}}^{(\exp )}$ and a local composition-dependent Δ_0_. We will refer to this as *semi*-*phenomenological approximation*. For the *g*-factor, a further simplification along this line is achieved by introducing constant values of Δ_0_ obtained by averaging the position-dependent values weighted by the squared wave function, which yields an explicit *effective Roth*-*Lax*-*Zwerdling formula* for the *g*-factor of a nanostructure, that is, Eq. (20) with the effective values of the parameters.
4. In the *off*-*diagonal* approximation, we include the full structure of the vb Hamiltonian *H*_v_, hence the full form of ${\mathscr{D}}$, but still neglect the *k*-dependent terms. In block notation,
  $${\mathscr{D}}=({E}_{{\rm{c}}}+{V}_{{\rm{p}}}+{a}_{{\rm{c}}}\,{\rm{Tr}}\eta ){\mathbb{I}}-(\begin{array}{ll}{H}_{{\rm{8v8v}}}^{(x)} & {H}_{{\rm{8v7v}}}^{(x)}\\ {H}_{{\rm{7v8v}}}^{(x)} & {H}_{{\rm{7v7v}}}^{(x)}\end{array}),$$
  where ${H}_{{\rm{8v8v}}}^{(x)}$, ${H}_{{\rm{8v7v}}}^{(x)}$, and ${H}_{{\rm{7v7v}}}^{(x)}$ are the position-dependent parts of the corresponding vb Hamiltonian blocks, formally obtained by setting *k* = 0 in Eqs (2b), (2c) and (2f), respectively, and ${H}_{{\rm{7v8v}}}^{(x)}={H}_{{\rm{8v7v}}}^{(x)\dagger }$. This yields a cb Hamiltonian strictly quadratic in *k* (and strictly equivalent to the original 8-band Hamiltonian up to the quadratic order), which corresponds to the usual notion of the effective mass equation in the parabolic approximation. Starting from this approximation, we go beyond the usual forms of the effective mass equations by introducing the full matrix structure of the vb-induced corrections.
5. The next step consists in including the self-consistent averages of the *k*-dependent terms in ${\mathscr{D}}$, that is
  22 $${\mathscr{D}}=({E}_{{\rm{c}}}+{V}_{{\rm{p}}}+{a}_{{\rm{c}}}\,{\rm{Tr}}\eta +\frac{{\hslash }^{2}}{2m^{\prime} }\langle {k}_{x}{k}_{x}+{\rm{c}}.{\rm{p}}.\rangle )\,{\mathbb{I}}-(\begin{array}{ll}{H}_{{\rm{8v8v}}}^{({\rm{av}})} & {H}_{{\rm{8v7v}}}^{({\rm{av}})}\\ {H}_{{\rm{7v8v}}}^{({\rm{av}})} & {H}_{{\rm{7v7v}}}^{({\rm{av}})}\end{array}),$$
  where ${H}_{{\rm{8v8v}}}^{({\rm{av}})}$, ${H}_{{\rm{8v7v}}}^{({\rm{av}})}$, and ${H}_{{\rm{7v7v}}}^{({\rm{av}})}$ are obtained from Eqs (2b), (2c) and (2f), respectively, by self-consistently replacing each product *k*_*i*_*k*_*j*_ with its average (denoted by 〈…〉) in the state of interest. In this way we go beyond the parabolic approximation. We will call this the *off*-*diagonal* + 〈*k*^2^〉 approximation. In this approximation the implicit character of Eqs (8a)–(8c) (the appearance of ${\tilde{H}}_{{\rm{c}}}$ and ${\tilde{H}}_{{\rm{v}}}$) is resolved to the leading order, that is, by replacing the two blocks of the Hamiltonian by the original *H*_c_ and *H*_v_.
6. The final approach in our sequence of approximations consists in approximating ${\tilde{H}}_{{\rm{c}}}$ and ${\tilde{H}}_{{\rm{v}}}$ in Eqs (8a)–(8c) self-consistently by renormalizing the effective mass and Luttinger parameters according to Eq. (9). The form of ${\mathscr{D}}$ is therefore the same as in Eq. (22) but with the renormalized parameters. We will refer to this as the *self*-*consistent effective mass equation*.

**Figure 1:**
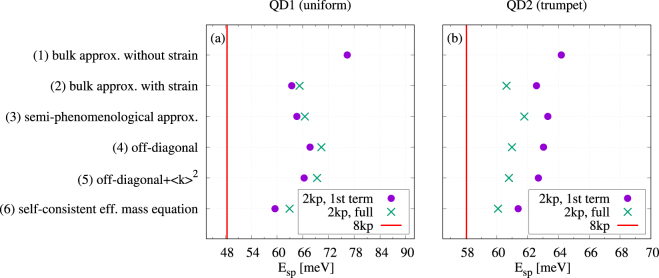
Energy splitting between the ground and first excited states at zero magnetic field for the sequence of approximations. Dots show the results obtained using only the first term in Eq. (12), while crosses represent the results from the full Hamiltonian. The red line shows the value obtained from the 8-band ***k*** · ***p*** calculation.

**Figure 2:**
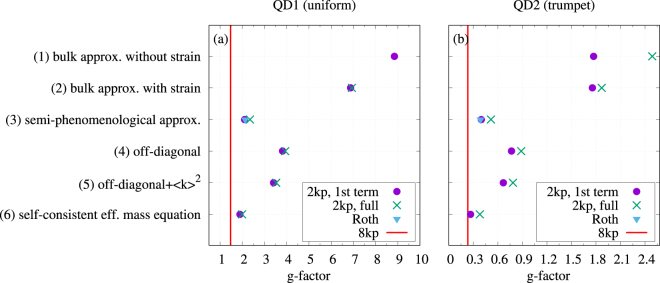
Ground state Landé factor for the sequence of approximations. Dots and crosses are defined as in Fig. 1. The triangle shows the result from the effective Roth formula. The red line shows the value obtained from the 8-band ***k*** · ***p*** calculation.

As a test of these approximations, we study the low-energy part of the spectrum of a self-assembled QD. We consider two models of a lens-shaped, self-assembled InAs/GaAs QD. In both models the QD has 24 nm diameter and 4.2 nm height but they differ in the composition profile: While the first model assumes a uniform composition with 100% InAs inside the QD, the second one has a more realistic trumpet-shape profile of InAs/GaAs composition with the InAs content defined by^45^ $C({\boldsymbol{r}})={C}_{{\rm{b}}}+({C}_{{\rm{t}}}-{C}_{{\rm{b}}})\,\exp \,[-\sqrt{{x}^{2}+{y}^{2}}\,\exp \,(-z/{z}_{{\rm{p}}})/{r}_{{\rm{p}}}]$, where we took *C*_b_ = 0.4, *C*_t_ = 0.8, *r*_p_ = 0.9 nm and *z*_p_ = 1.4 nm. In both cases the QD is placed on a 0.6 nm wetting layer (WL), which in the case of the first dot contains 100% and in the latter one 40% InAs. The two figures of merit that we will investigate here are the energy splitting Δ*E*_*sp*_ between the ground (“*s*-shell”) electron state and the lowest excited (“*p*-shell”) state at zero magnetic field (Fig. 1) and the ground state *g*-factor, extracted from the leading order (linear) term of the Zeeman splitting of the ground-state doublet at low magnetic fields (Fig. 2). In both cases we show in the plots the values obtained using only the first, usual term in Eq. (12) (points) and those from the full equation, including also the non-standard second term (crosses).

As shown by the results in Fig. 1, the effective mass methods typically fail to reproduce the *s*-*p* shell splitting which, in fact, belongs to the most fundamental quantitative characteristics of a QD system. What matters here is the renormalization of the ***k*** · ***p*** parameters according to Eq. (9), hence the self-consistent equation (method (6) from our series of approximations) is able to produce a result that is closest to the correct value. For the uniform QD, Fig. 1(a), the disagreement is still at the level of 30%, while for the more realistic model with the trumpet-shape composition profile Fig. 1(b), the effective mass result is much more exact. The 30% discrepancy is consistent with the estimate of the error induced by the linear truncation in Eq. (6). The much higher precicision in the case of the smooth trumpet-shape composition may be due to the fact that terms of higher order in *k* in an envelope-function theory for an inhomoheneous system are not just “non-parabolic” but also induce higher order derivatives of system parameters, to which the uniform QD model with abrupt compositional boundaries is obviously much more sensitive. One can understand why approximation (6) is particularly suited for relatively correct modeling of this particular spectral feature by noting that the *s*-*p* shell splitting is related to the in-plane excitation of the QD, for which the value of the effective mass plays a crucial role. The renormalization described by Eq. (9) is an important correction to this parameter. On the contrary, including the non-parabolicity correction, either by self-consistently adding the average *k*^2^ terms in the ${\mathscr{D}}$ operator, as in the approach (5), or by using a phenomenological value of *E*_g_, as in the approach (3), mostly reflects the impact of the strong confinement in the growth direction, which shifts the lowest shells rigidly, hence does not affect the *s*-*p* splitting considerably. It is found also that including the full matrix structure of *H*_v_ in ${\mathscr{D}}$ (approximation (4)) does not bring any clear advantage by itself. In addition, it turns out that correcting the effective mass equation by the second term in Eq. (12) has an opposite effect on the two models: it decreases the accuracy for the homogeneous QD, while it improves it (to a larger degree) for the trumpet-shape model. We were not able to find a plausible explanation of this fact. Interestingly, the correction stemming from this term is roughly the same for a given QD structure in each approximation.

The accuracy of various approximations to the effective mass equation is different when the *g*-factor is considered (Fig. 2). Here the approximations (4–6), where the full matrix structure of the vb is included, yield much more accurate results than the diagonal approximations (1) and (2). As one can see in Fig. 2, the self-consistent model (6), with self-consistently renormalized parameters, again provides the most accurate results in the systematic series of approximations. The discrepancy is about 60% for the uniform QD model (Fig. 2a) while the result for the model with a trumpet-shape composition profile (Fig. 2b) is overestimated by a factor of 2. In the case of the *g*-factor, including the part of non-parabolicity that is captured by our self-consistent approach improves the accuracy to some extent but is not critical. Also the correction provided by the second term in Eq. (12) is of much less importance than it was for the spectrum, with a larger relative contribution in the trumpet-shape model. In both models, it turns out to lower the accuracy. The large error in the values of the *g*-factor apparently exceeds the estimate of the effect of truncating Eq. (6). In fact, however, the numerical values should be referred to the bulk value of −15, which is nearly entirely compensated by confinement and strain effects. Therefore, the mismatch is actually of only a few percent.

It seems interesting that accurate systematic modeling of the Zeeman splitting in an effective mass equation requires the full form of the operator ${\mathscr{D}}$. This means that the standard Eq. (19) is not very useful if the *g*-factor is calculated by Löwdin perturbation theory including only the diagonal contributions to the vb structure (in particular, the hh-lh splitting induced by the axial-strain-related *b*_v_ terms): for our two structures the approach (2) overestimates the Zeeman splitting by an order of magnitude. We were able to trace this back to the important role played by the off-diagonal terms proportional to the *b*_v_ deformation potential in the correct alignment of valence bands: including axial strain contributions only in the diagonal terms produces wrong vb alignment (in terms of band splitting and the sign of the band offsets between the QD and the barrier). As a consequence, completely neglecting these subtle details of the vb, as done in the semi-phenomenological approximation (3), leads to a result which is only slightly worse than the most exact one when compared to the full 8-band model. Here the lh-hh splitting and band offsets are completely washed out by setting a single constant value for the fundamental band gap and only the spin-orbit (Γ_7_) band splitting is position dependent. The result becomes even more accurate if the latter is also replaced by a single number obtained from spatial averaging of the standard bulk values (black triangles in Fig. 2). Thus, our numerical results support the commonly used and very convenient Roth-Lax-Zwerdling formula for estimating the electron *g*-factor.

## Conclusions

In this paper we have proposed a systematic derivation of a cb effective mass equation from the 8-band ***k*** · ***p*** Hamiltonian. We have shown that this derivation develops into a systematic series of approximations that differ in the way the vb is represented in the final equation. Possible approximations range from using a set of fixed band gap parameters to a full matrix structure with self-consistent non-parabolicity corrections and parameter renormalization. We have assessed the accuracy of the approximations in calculating selected spectral and spin-related characteristics for a self-assembled QD system within two models of the composition profile.

We have shown that a quantitatively correct description of the lowest sector of the electron spectrum, which involves intraband dynamics and therefore relies primarily on the accurate modeling of the effective mass, requires a self-consistent renormalization of the Hamiltonian parameters that goes beyond the second order Löwdin perturbation. The accuracy is also improved by accounting for cb non-parabolicity by self-consistently including terms of higher order in the electron momentum. When studying the Zeeman splitting of the ground electron level we found that the most accurate value of the *g*-factor is obtained within the systematic scheme only after including the full structure of the vb Hamiltonian in the equation. Here, again, including non-parabolicity corrections improves the accuracy of the result. Surprisingly, the values obtained from the resulting rather complicated equation can be reproduced by a simple diagonal model with a fixed value of the fundamental band gap, which supports the use of the effective Roth-Lax-Zwerdling formula for a nanostructure. The accuracy of the latter is remarkable, given its simplicity. The effective mass equations reproduce 8-band ***k*** · ***p*** value of the electron *g*-factor for the QD ground state within a factor of 2, which may seem disappointing. One should take into account, however, that the value of the *g*-factor for bulk InAs is about −15, which is nearly entirely compensated by confinement and strain effects. The absolute mismatch, which is on the order of 0.1, is a tiny fraction of the original value (that is, the compensation is quantitatively reproduced) but yields a large relative error when compared to the very small final value.

In general, we have shown that the effective mass equation offers a limited accuracy when modeling the lowest-energy sector of the electron spectrum in a self-assembled QD, unless it is extended to a rather complicated, inconvenient and computationally expensive form. In particular, an equation that is rigorously derived from the ***k*** · ***p*** theory strictly up to order *k*^2^ (which may correspond to the most usual notion of the *effective mass theory*) quantitatively fails in all respects. The more accurate self-consistent equation proposed in this paper is not particularly transparent and does not even allow a separation into kinetic and Pauli terms. On the other hand, we have shown that the electron *g*-factor can be estimated by a very simple version of the effective mass theory, thus justifying the phenomenological Roth-Lax-Zwerdling formula for a nanostructure. Our derivation highlights the correspondence between the BenDaniel-Duke and Gora-Williams-Bastard orderings of the kinetic term, both emerging as different approximations within a general scheme, and yields a generalized Rashba coupling including inhomogeneity and strain effects. This confirms once more that the effective mass equation, even though not perfectly accurate, can be very useful from the conceptual point of view and often provides valuable physical insight.

### Data availability

The datasets generated during and/or analysed during the current study are available from the corresponding author on reasonable request.
